# Sustainability at the Edge of Chaos: Its Limits and Possibilities in Public Health

**DOI:** 10.1155/2013/801614

**Published:** 2013-08-24

**Authors:** Christopher G. Hudson, Yvonne M. Vissing

**Affiliations:** ^1^School of Social Work, Salem State University, 352 Lafayette Street, Salem, MA 01945, USA; ^2^Department of Sociology, Salem State University, Salem, MA 01970, USA

## Abstract

This paper critically reviews the expanding literature on applications of sustainability to healthcare policy and planning. It argues that the concept has been overgeneralized and has become a buzzword masking disparate agendas. It ignores the insights of the newest generation of systems theory on complex systems on the ubiquity of far-from-equilibrium conditions. Yet, a central meaning often ascribed to sustainability is the level continuation of healthcare programs and their institutionalization. Sustainability is only coherent in health care when it is more narrowly delimited to involve public health and treated as only one of several evaluative criteria that informs not only the continuation of programs but more often their expansion or contraction as needs dynamically change.

## 1. Introduction

**Figure 1:**
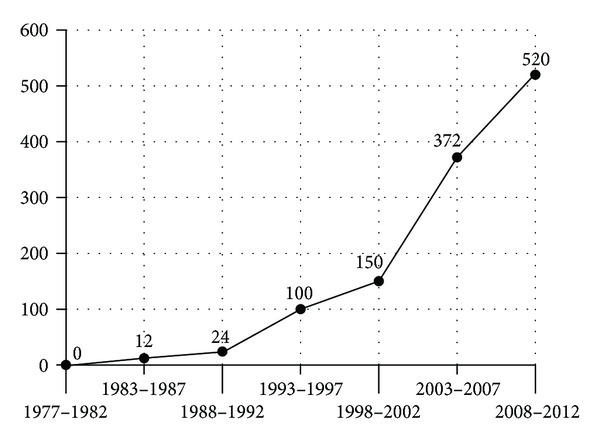
Articles published on health and sustainability 1977 between 2012. Source: Google Scholar (2013).

 Some commentators complain that sustainability has become a buzzword, even an oxymoron [1], and argue that the “sustainable development” of healthcare policies and programs is a logical impossibility. Whatever the merits of these arguments are, there has been an exponential growth of interest in the application of sustainability to healthcare in the recent years (see Figure 1). Out of 56,500 articles published between 1977 and 2012 with “sustainability” in the title, 1,178 have focused on its relevance to health, healthcare policies, or programs [2]. The growth in scholarly interest in these applications is part of a broader interest in grounding healthcare policy and planning in social ecology, and in incorporating the concerns and insights of the environmental movement [3]. The sources of this interest are diverse; however, the increasing vulnerability of healthcare organizations is no doubt fueling an interest in organizational survival, even more than the continued development, fine-tuning, and targeting of healthcare services. The promotion of sustainability is conceptualized by some as a strategy for institutionalization [4], divorcing the concept from its original and narrower focus on the protection of environmental resources, renewability, and intergenerational equity.

 This paper critically reviews selected applications of sustainability in healthcare, regarding both their limitations and possibilities. It does this, in part, by drawing on insights from complex systems theory which has introduced a paradigmatic shift towards thinking about policy systems as necessarily operating at far-from-equilibrium conditions, specifically, in a dynamical regime referred to as the “edge of chaos”, where systems adapt and thrive only when there is a fine-tuned balance between periodic and chaotic processes [5–8]. It is an open question what sustainability can mean when equilibrium conditions are the exception rather than the rule as is traditionally assumed. This paper, thus, calls for a more limited use of sustainability that focuses on public health and well-being, rather than policies and programs and their survival. When used with health programs, the paper argues that sustainability should only be used as one of a range of evaluative criteria and their tradeoffs, including innovation, adaptability, responsiveness, equity, effectiveness, efficiency, and efficacy. Otherwise, the ideal of sustainability degenerates into a strategy of indiscriminate organizational survival and goal displacement.

 Given the diversity of applications of and approaches to the study of sustainability that originate from a wide variety of disciplines, a systematic review of the larger body of this literature, including the 1,178 articles mentioned above and their supporting research, is not attempted here and may not even be possible. Rather, what is reported are the results of a thematic or critical review of the major approaches to its conceptualization and selected applications and implications that emerge from it. The forty-nine (49) sources selected for inclusion in this paper were chosen not so much as a representative sample, but more for their ability to illustrate the major arguments and approaches to the subject.

## 2. Background

 Beginning with Thomas Malthus' ominous prediction in the late eighteenth century that food supply would invariably lag behind population growth [9], there have been continuing problems involving the destructive impact of industrialization on both social and physical environments. Carson's classic *The Silent Spring* [10] about environmental degradation is often cited as the single most important stimulus for the environmental movement which has sought to protect the physical environment from unrestrained growth and to assure intergenerational equity in the extraction of its resources. It was the environmental movement, launched in the 1960s, that set the stage for periodic international commissions and conferences which have introduced and attempted to refine the notion of sustainability as a guiding principle governing economic growth, or, at least, stability. Among the most notable of these was the work of the World Commission on Environment and Development, established in 1983 by the United Nations. This commission, headed by Gro Harlem Brundland, issued a report that introduced the ideal of “sustainable development,” or the notion that human activity should “meet the needs of the present without compromising the ability of future generations to meet their own needs” [11]. Since then, debate has shifted from a focus on environmental issues, through a shared emphasis on poverty alleviation, to more recent, prioritizing improvements in social well-being, for instance, through improved healthcare, while protecting the environment [12].

 Sustainability, thus, has taken on a range of meanings that are exemplified by its many proposed definitions. Whereas one dictionary notes that it is the ability “to be maintained at a certain rate or level” [13], other definitions emphasize public policy approaches, one of which defines sustainability as the “satisfaction of basic economic, social, and security needs now and in the future without undermining the natural resource base and environmental quality on which life depends” [14]. In the arena of healthcare, sustainability has been defined as the “continuation of programs” [4] and is theorized to be achieved through two sustainability processes of *“routinization* and *standardization”* [15]. Such approaches suggest that sustainability has been overgeneralized and redefined simply as organizational survival, divesting it from its focus on environmental protection and minimizing the classic ecological dilemma known as the tragedy of the commons. This is the possibility that any time environmental resources are treated as economic externalities and are unpriced, they tend to be overused and degraded, in a similar manner as that of animals when they strip an unregulated common area in which they are permitted to freely graze.

## 3. Sustainability in Healthcare

 This section considers the meanings ascribed to sustainability specifically in healthcare. This ideal has been promoted both within healthcare institutions, particularly community health within social development contexts, and within the field of alternative medicine. The Alliance for Natural Health (ANH) claims to have first defined sustainable healthcare in 2006 in the journal *Nutrition Practitioner* as follows:

A complex system of interacting approaches to the restoration, management and optimisation of human health that has an ecological base, that is environmentally, economically and socially viable indefinitely, that functions harmoniously both with the human body and the nonhuman environment, and which does not result in unfair or disproportionate impacts on any significant contributory element of the healthcare system [16].

Conceptual definitions such as the above, as reasonable as they may be, fail to capture the many operational decisions that are necessary for the implementation of this ideal. At a minimum, these decisions include fundamental questions of what, when, why, how, and who. Answers to the question of what is to be sustained range from population health, to healthcare policy systems, to funding levels, and to particular types of programs and treatments. Are these to be sustained for a defined period, on a continuous and indefinite basis, until they are shown to be ineffective, or until the need changes or better programs and treatments are developed? Definitions of ideal and tolerable levels of health and disease inform the question of why; available technologies impact questions of how sustainability is sought; and moral, social, political, and spiritual values are central to answering questions of whose health is to be sustained and who should sustain it.

 Although one might expect that the focus of healthcare sustainability would be the health of the public, this is paradoxically the least frequent area of its applications. Although many researchers assume public health as an overarching value, there are others who are more committed to the maintenance or expansion of existing medical services regardless of their public health outcomes. There is a growing body of research that supports the notion that existing medical practices may be less important to the overall health of the public than broader social conditions, for example, socioeconomic conditions, patterns of exercise, nutrition, sanitation, and the like. With the exception of the work of the ANH which is concerned with the “restoration, management, and optimization of human health,” most applications in healthcare have been predominately concerned with maintaining funding levels and systems, or sustaining particular programs or services. Some of the commentators who emphasize the sustainability of policies or programs cite the importance of sustainability of healthcare financing and benefits. For example, Shediac-Rizkallah and Bone [4] note that one of the several important categories of benefits to be sustained is that of the “maintenance of health benefits achieved through an initial program.” Yet, too often an initial health benefit turns out to be better achieved through alternative programs or treatments. A case in point involves the initial benefits of public psychiatric hospitals that can now be better achieved in community mental health settings using alternative services such as intensive case management or psychiatric rehabilitation. 

 The rising costs of healthcare in the USA and other western nations have generated considerable efforts to contain and stabilize funding patterns. It is in this context that sustainability has come to mean for many the sustaining of national healthcare expenditures. In the USA this has meant the increasing introduction of capitation and other managed care techniques into the funding and oversight of healthcare delivery, and, increasingly, within the public Medicaid and Medicare programs. Within the Medicare program, there have been efforts to implement a “sustainable growth rate” formula to control physician payments, but with limited success [17]. In Europe, Pammolli et al. [18] argue that the central need of many European governments is to assure pluralistic systems in which a balanced mix of public and private funding sources assures a “balance of sustainability and access.” In Canada, Guyatt and colleagues [19] present a set of recommendations to assure the stability of public health funding, noting that “by sustainability, we mean having mechanisms in place to ensure that Canadians, irrespective of their ability to pay, will have continued access to prompt, technologically current, competent and compassionate healthcare that addresses the full range of their health needs.” In an analysis of the Swedish welfare system—“Market reforms in healthcare and sustainability of the welfare state: lessons from Sweden”[20]—Diderichsen concludes that inequities and the resulting lack of sustainability in healthcare provision are functions of the way healthcare deals with inequities in health due to disparities resulting from living conditions versus those resulting from ill health.

 A literature has emerged on the sustainability of local community health programs. Some have taken a broad view of this as including the problem of diffusing successful ideas and innovations [21]. Shediac-Rizkallah and Bone [4] report on research that suggests that the sustainability of local health initiatives derives from the following: (i) project design and implementation factors; (ii) factors within the organizational setting; and (iii) factors, involving local political buy-in, in the broader community environment. These authors argue that, overall, sustainability is best thought of as involving program continuation even more so than program institutionalization. One analysis of the implementation of mental health programs in Australia focuses on their financial sustainability, noting that “our aim was to provide insights into the economic credentials of both interventions, as well as to comment on their sustainability, both financial and in terms of the broader issues of workforce requirements and consumer acceptance” [22]. Unfortunately, too often, the concern for the institutionalization, continuation, or sustainability of health programs, especially for their financial viability, eclipses attention to their effectiveness and responsiveness to public health needs. Such alternative criteria may dictate either the expansion of effective and needed programs or the phasing out of ineffective programs, and, perhaps less frequently, their level continuation.

 Advocates for the use of sustainability as a central yardstick for guiding healthcare policy and planning have occasionally sought to operationalize and develop measures for it [23]. These include indicators, benchmarks, auditing and accounting procedures, assessment protocols, and various types of reporting systems. These procedures are still evolving, and there are no universally agreed upon protocols. Their development will no doubt be contingent not only on better conceptualization of sustainability, especially, as one of the many important criteria for the evaluation of healthcare programs and interventions, including their efficacy, effectiveness, responsiveness, and adaptability. This is not to suggest that such advocates of sustainability assessment are oblivious to alternative criteria. For example, it has been pointed out that, in ecological economics, although it has the explicit goal of sustainable scale in contrast with continuous growth, fair distribution and efficient allocation are secondarily valued. It might be argued that the health of the public requires a greater priority to be given to efficacy, effectiveness, responsiveness to public health needs, and equitable distribution of healthcare resources. 

## 4. The Edge of Chaos

 The focus of this paper is on the meaning of sustainability in public health under inherently unstable and changing conditions, involving the phenomenon known as the “edge of chaos.” The newest generation of systems theory, variously referred to as the complexity sciences, complex adaptive systems, or nonequilibrium theory, has among its central themes the ubiquity of far-from-equilibrium systems and the resulting limitations in their predictability. Complex systems theory is built on a diverse range of metaphors, hypotheses, mathematical theorems, and modeling and simulation techniques. Among the major component theories are those concerned with fractals, cellular automata, self-organization, chaos, bifurcation, and autopoiesis (see [24]). An offshoot of several of these is the notion of the “edge of chaos”, a term first coined by the mathematician Doyne Farmer (see [6]) to describe a transition phenomenon first discovered by the Computer Scientist Langton [25]. It refers to dynamical processes that incorporate both elements of periodicity and randomness, or, sometimes, chaos as defined by the mathematics of chaos theory. It was Roger Lewin who popularized the notion and commented the following:

The edge of chaos is where information gets its foot in the door in the physical world, where it gets the upper hand over energy. …Being at the transition point between order and chaos not only buys you exquisite control—small input/big change—but it also buys you the possibility that information processing can become an important part of the dynamics of the system [8, page 51].

It should be noted that complex systems theory is at root an extensive array of mathematical and modeling techniques. These, however, are only rarely used by either professionals or even academic researchers in the study of healthcare systems. Typically, most applications have involved the conceptual and metaphoric renditions of these ideas. Few professionals have the requisite quantitative skills, letting alone the extensive data required for such applications. Many have criticized the metaphorical treatment of the subject, pointing out many misunderstandings and misapplications of the fundamental ideas. Nonetheless, the metaphorical level is an important *starting point* for this work. According to the Philosopher of Science, Max Black, “most scientific models are “systematically developed metaphors” (cited in [26]). Even in the hard sciences, metaphors are routinely used (consider Bohr's solar system metaphor of the atom), but then they are operationalized, quantified, and tested [24].

 Although popularizations of the notion of the edge of chaos have relied heavily on intuitively understandable metaphors, several scientists, most notably the Biologist Stuart Kauffman, have mathematically operationalized the idea. He emphasized the degree of *connectivity* of the component parts of living systems, classifying systems in terms of the ratio of internal interconnections (*k*) to total units (*n*), or *k/n*. When *k* is very small in relation to *n,* a “sub-critical system” develops, which exhibits little adaptability and an excessively steady state. When the ratio of *k* to *n* increases beyond a certain threshold, the system enters the “edge of chaos” in which there are elements of periodicity as well as chaos and randomness and maximum adaptability. Finally, if the number of interconnections (*k*) approaches or exceeds the number of component parts (*n*), a system will develop which is “supercritical,” highly unstable, and best described and modeled using the mathematics of the chaos theory [27]. Kauffman argued that the evolution and the adaptability of both individuals and species thrive at the edge of chaos. Researchers who study such systems have often used a statistical index referred to as the Lyapunov coefficient to measure the edge of chaos. When this coefficient is negative, the system is subcritical; when it is close to zero, it enters the “edge of chaos” regime; and when it substantially exceeds zero, the system becomes supercritical [28]. 

 Several authors have shown that the “edge of chaos” is necessary for or conducive to self-organization [5–8], but probably not a sufficient condition. For instance, the Nobel Laureate Ilya Prigogine points out that many natural systems spontaneously organize themselves while being on the borderline between order and chaos [29]. He argues that the maintenance of organization in nature cannot be achieved by “central management,” but only through self-organization: “Self-organizing systems allow adaptation to the prevailing environment…and makes the system extraordinarily flexible and robust against perturbations from outside conditions” [30, page 71]. Elsewhere Prigogine points out that “When an open system is far from equilibrium, under the influence of a driving force, random fluctuations either internal or external to the system are amplified within the system, involving system-wide communication, and experimentation occurs with possible new configurations” [31].

 For the reasons first articulated by Kauffman and Prigogine, far-from-equilibrium conditions and the more circumscribed edge of chaos dynamic are viewed as a fundamental threat to sustainability and an opportunity for problem-solving and adaptation, among both individuals and larger organizational and community systems. For instance, the Psychologist Richards argues that the ability to function at the edge of chaos is one of the most important conditions for creativity and effective problem-solving [7]. Because chaotic processes have been mathematically demonstrated to never repeat themselves [32], they represent an endless source of novelty. 

 Applications of the complex systems theory and the edge of chaos dynamic abound, not only in the sciences but also in business, human services, and healthcare. Hock [33] writes extensively in business on “chaordic” organizations that thrive at the edge of chaos. Guastello [34] reviews applications in psychology.

Several other authors apply these insights to healthcare [35, 36]. Papadopoulos et al. [37] hypothesize that problems of waiting lists in UK's National Health Service can be understood through the lens of the edge of chaos. More extensive reviews of applications of the edge of chaos to healthcare and/or public health than are possible here have been published elsewhere. Lindberg et al. [38], for example, present two cases involving a clinical case of dissociative identity disorder and the reorganization of a healthcare management team, each of which is used to illustrate the efficacy of moving from problem-solving approaches that emphasize command and control with predictable (sustainable) structures to ones that focus on patterns of change, with divergent and even paradoxical elements, and which draw on both integrative and divergent forms of intelligence. After reviewing a variety of applications of complex systems thinking in public health, Trochim et al. [39] use concept mapping, a systems-based research tool, to clarify the interrelationships among key systems concepts used by the participants of such several public health projects. Among their conclusions is that public health planners need to recognize “that planning and evaluation are not yet sufficiently systemic and that planning should be continuous and adaptive, with constant feedback among planning, action, and evaluation.” It is, thus, clear that although applications of complex systems and edge of chaos notions in public health have dramatically expanded in the recent years, they clearly have not entered the mainstream where the focus remains on the sustainability of existing structures and the maintenance of equilibrium rather than the dynamical processes and the facilitation of adaptive change.

The rarity of truly closed, predictable, and unchanging systems effectively balanced in equilibrium—whether population health or healthcare policies are considered—has been a common theme among complex systems theorists and researchers. If true, this suggests that sustainability may be a chimera since there are forever changes requiring the elimination or expansion, and only occasionally the level sustenance of any particular healthcare state or system. The notion that creative problem-solving thrives at conditions involving the edge of chaos suggests that the underlying ideals of healthcare promotion may be more effectively achieved through a more delimited application of the notion of sustainability, and a more nuanced or complex understanding of changing healthcare systems. However, there are a variety of other limitations of the notion of sustainability, to which we will now turn. 

## 5. Limitations of Sustainability

 The scarcity of serious critique of sustainability may simply reflect a pervasive endorsement of both the ideals and the practical implications of this notion. Nonetheless, there have been several criticisms of sustainability, both in general and in healthcare in particular. 

 Most of these critiques derive from the complaint that sustainability is rhetoric, a buzzword, a reification, or a nominalization of dynamic processes into a kind of static “totalizing doctrine that subsumes critical thinking” [40]. An anonymous Wikipedian author points out that, at one extreme, the term is used as a feel-good buzzword, and, at the other extreme, it is an important but unfocused concept like “liberty” or “justice” [41]. 

 Another author contends that, as a popular term, sustainability is a “signifier onto which so many different aspirations and agendas have been projected, that it doesn't really mean anything any more” [42]. This comment points to the second major critique in the literature, namely, that sustainability covers and obscures a variety of competing agendas, from both the right and the left. One author, Ricketts [43], argues that competing agendas from within the environmental movement have created pressures not to define sustainability. 

 Others contend that sustainability is often applied in inappropriate areas such as housing, urban development, and healthcare. Marcuse [44] points out that many lousy programs are sustainable, and, when sustained, they may limit the scaling up of new and innovative programs, including new healthcare treatments and other interventions. As such, sustainability may become a rationale for maintaining the *status quo*. In its application to urban environments, Marcuse argues that, as a way of promoting an unjust *status quo*, sustainability may be used to suggest that everyone has common interests when there are very real conflicts of interests. Luke [45] argues that the technocratic tools of ecomanagerialism, ecocommercialism, and ecojudicialism are means to “manage and mitigate the damage inflicted upon nature” (page 101). The effort to reconcile sustainability with continued development—as “sustainable development”—is cynically recast as a kind of “sustainable degradation,” as a means of attempted but failed maintenance of an unworkable *status quo *[45]. 

 The diverse agendas that are invested into the ideal of sustainability and their critique are epitomized by two diametrically opposing viewpoints. On one hand, some deep ecologists argue that sustainability is “too human centric” [46], unsupportive of nature and the many nonhuman species. On the other hand, some complain that sustainability is too “antiprogress,” that it is “a pernicious and corrosive doctrine that has survived primarily because there seems to be no alternative to its canon,” and that it is a “malign philosophy of misanthropy, low aspirations, and restraint” [47]. Such people attack the ideal of sustainable development from a conservative, free market perspective, arguing for the abundance of natural resources and human technological ingenuity.

 Insights from the study of complex systems, particularly the chaos theory, point to one of the most fundamental limitations of sustainability, namely, the ubiquity of change, whether gradual or rapid, and the notion that states of equilibrium are the exception and that, even when they exist, they are only temporary with regular transitions between multiple equilibria. The ongoing coevolution of human and nonhuman organisms, including bacteria, as well as the constant development of new healthcare delivery systems, the discovery of more effective treatments, and the discontinuation of antiquated treatments all undermine the usefulness of sustainability as overarching criteria or yardstick for the guiding of health care policy and planning.

## 6. Possibilities

 The ideals at the historical core of the idea of sustainability represent its most important possibilities. These include the imperative of the protection of the natural environment, assurance of its renewability, and the commitment to intergenerational equity. These are values that the most endorse. Increasingly, sustainability has been generalized to be also applied to a wide range of social and economic systems, their protection, and their renewability. It is in this arena that controversies about the maintenance of the* status quo*, the protection of ineffectual systems, the stifling of innovation, and the development of successful healthcare treatments and practices inevitably arise. The practice of sustainability assessments [48], that is, the examination of what is needed or missing for the survival of a healthcare funding system or a local health program, is a needed exercise, but only when it has been first established that the healthcare practice is itself effective and responsive to public needs and has been implemented at an appropriate scale. 

Perhaps the more important challenge involves assessing the barriers to and the requirements for sustaining optimal health and well-being, and this involves grappling with the difficult tradeoffs between secondary medical and rehabilitative care and interventions designed for the promotion of health, whether these involve nutrition, inoculations, sanitation, exercise, abatement of violence, and cultural and economic health of a population. For example, to the extent that malaria can be eradicated through inoculations, mosquito nets, spraying, better sanitation, or some combination of these, then secondary medical care can be minimized. It is the health of human beings, as they are situated in their physical and cultural environments, that should be the primary focus of such sustainability assessments rather than particular packages of treatments. 

Because of the lack of any agreed-upon definition of sustainability, as is documented in this paper, it simply has not been possible for healthcare researchers to empirically evaluate the outcomes of applying policies based on diverse conceptualizations of sustainability, as much as the plausibility of the various limitations and possibilities might be argued by their proponents and detractors. The diversity of definitions, theoretical approaches, and underlying values points to one of the overriding conclusions of this paper, to which we will now turn.

## 7. Conclusions

 The central argument of this paper is that the overgeneralization of the ideal of sustainability, especially as it has come to be understood as the institutionalization of healthcare funding and programs, has obscured the underlying ideals that motivated the initial development of this concept. Sustainability makes for effective rhetoric, but as rhetoric it both expresses and hides a variety of competing and even incompatible agendas, involving the continued growth, institutionalization of programs, and the minimalist agendas of deep ecologists. For the concept to have meaning, it is essential that it be more sharply delimited. One way to do this in healthcare is through focusing it on population health. But it should also be delimited by a recognition that sustainability is only one from among many important evaluative criteria that should inform healthcare policy and planning; others include effectiveness, efficacy, responsiveness, and equity, to mention a few. 

 The needs of a population's health and the application of the range of these criteria for evaluating various healthcare alternatives should be the central considerations in both sustainability assessments and, more broadly, healthcare planning. The conclusion of any such sustainability assessment and health planning may be to innovate and perhaps disseminate a given option, if it is proven to be optimal, and, when scaled up to an appropriate degree, to sustain it, or even to phase it out if needs diminish or more effective alternative interventions emerge. One might argue that a broader definition of sustainability should include all of the foregoing possibilities, but this would divest the concept of its usefulness as a practical evaluative principle and criterion. Rather, sustainability is coherent only when it is understood more narrowly in terms of the ongoing support and flourishing of a universally-valued outcome, such as human health, rather than any particular means for bringing it about.
